# A new web-based method for automated analysis of muscle histology

**DOI:** 10.1186/1471-2474-14-26

**Published:** 2013-01-16

**Authors:** Cordula Pertl, Markus Eblenkamp, Anja Pertl, Stefan Pfeifer, Erich Wintermantel, Hanns Lochmüller, Maggie C Walter, Sabine Krause, Christian Thirion

**Affiliations:** 1 Laboratory of Molecular Myology, Friedrich-Baur-Institute, Department of Neurology, Ludwig-Maximilians-Universität München, Marchioninistraße 17, 81377, Munich, Germany; 2 Institute of Medical and Polymer Engineering, Technische Universität München, Munich, Germany; 3 S.CO LifeScience GmbH, Munich, Germany; 4 Institute of Genetic Medicine, Newcastle University, Newcastle upon Tyne, United Kingdom; 5 SIRION BIOTECH GmbH, Planegg/Martinsried, Germany

**Keywords:** Duchenne muscular dystrophy, *md*x mouse, Histological muscle fibre analysis, Standardised and automated quantitative analysis, Minimal Feret’s diameter

## Abstract

**Background:**

Duchenne Muscular Dystrophy is an inherited degenerative neuromuscular disease characterised by rapidly progressive muscle weakness. Currently, curative treatment is not available. Approaches for new treatments that improve muscle strength and quality of life depend on preclinical testing in animal models. The *mdx* mouse model is the most frequently used animal model for preclinical studies in muscular dystrophy research. Standardised pathology-relevant parameters of dystrophic muscle in *mdx* mice for histological analysis have been developed in international, collaborative efforts, but automation has not been accessible to most research groups. A standardised and mainly automated quantitative assessment of histopathological parameters in the *mdx* mouse model is desirable to allow an objective comparison between laboratories.

**Methods:**

Immunological and histochemical reactions were used to obtain a double staining for fast and slow myosin. Additionally, fluorescence staining of the myofibre membranes allows defining the minimal Feret’s diameter. The staining of myonuclei with the fluorescence dye bisbenzimide H was utilised to identify nuclei located internally within myofibres. Relevant structures were extracted from the image as single objects and assigned to different object classes using web-based image analysis (MyoScan). Quantitative and morphometric data were analysed, e.g. the number of nuclei per fibre and minimal Feret’s diameter in 6 month old wild-type C57BL/10 mice and *mdx* mice.

**Results:**

In the current version of the module “MyoScan”, essential parameters for histologic analysis of muscle sections were implemented including the minimal Feret’s diameter of the myofibres and the automated calculation of the percentage of internally nucleated myofibres. Morphometric data obtained in the present study were in good agreement with previously reported data in the literature and with data obtained from manual analysis.

**Conclusions:**

A standardised and mainly automated quantitative assessment of histopathological parameters in the *mdx* mouse model is now available. Automated analysis of histological parameters is more rapid and less time-consuming. Moreover, results are unbiased and more reliable. Efficacy of therapeutic interventions, e.g. within the scope of a drug screening or therapeutic exon skipping, can be monitored. The automatic analysis system MyoScan used in this study is not limited exclusively to dystrophin-deficient mice but also represents a useful tool for applications in the research of other dystrophic pathologies, various other skeletal muscle diseases and degenerative neuromuscular disorders.

## Background

Duchenne muscular dystrophy (DMD) is an inherited, progressive disorder affecting striated and cardiac muscles. It is caused by mutations in the dystrophin gene [1,2], one of the largest genes in the human genome. This gene encodes a large cytoskeletal protein called dystrophin that links the cytoskeleton to the extracellular matrix [3-5]. DMD is the most prevalent X-linked recessive neuromuscular disorder, affecting 1 in 3500 male children. There is still no curative treatment available. Current treatments for DMD are symptomatic and significantly improve longevity and quality of life but hardly prevent loss of muscle function [6]. Short-term prednisone treatment is recommended to preserve motor functions in ambulant DMD patients [7].

A naturally occurring dystrophin-deficient mutant was first described in 1984 in a colony of C57BL/10 mice (C57BL/10ScSnJ) [8] and has since been referred to as the “*mdx* mouse”. This mouse, now called C57BL/10ScSn-Dmdmdx/J, is among the most frequently used mouse models used in basic research. It carries a point mutation in exon 23 of the mouse dystrophin gene introducing a premature stop codon, which leads to the loss of full-length dystrophin [8]. In the *mdx* mouse the acute onset of myofibre necrosis occurs around 21 days of age. The acute onset of dystrophic pathology with high levels of necrosis provides a very sensitive assay to specifically evaluate some effects of therapeutic interventions designed to prevent or reduce myofibre necrosis. Normal myofibres show peripheral nuclei, intact sarcolemma and non-fragmented sarcoplasm. Necrotic muscle is characterised by infiltrating inflammatory cells, hypercontracted myofibres and degenerating myofibres with fragmented sarcoplasm. Regenerating rodent muscle subsequently displays myofibres with internal nuclei (regenerated myofibres). Internal nuclei are the feature of regenerated rodent muscle. In other species, including humans, the nuclei become peripheral and they are not necessarily a marker of regeneration [9,10]. Cumulative skeletal muscle damage in young *mdx* mice comprises of active myofibre necrosis as well as areas of subsequent regeneration (new myofibres). Myonuclei of newly regenerated myofibres of *mdx* mice remain in an internal location for about 50–100 days. Subsequently, 3–4% of the myonuclei move to a peripheral subsarcolemmal position [11]. Myofibre size can be determined by the analysis of the cross sectional area. Values for the myofibre size may be distorted by the orientation of the sectioning angle. This problem can be avoided by measuring the minimal Feret’s diameter defined as the minimum distance of parallel tangents at opposing borders of the muscle fibre [12]. Dystrophic muscle typically displays a higher variability of the muscle fibre diameter as compared to wild-type muscle. The variance coefficient (VC) of all muscle fibres’ minimal Feret’s diameters of a given muscle cross-section provides a numerical value of the myofibre size variability [12].

Subsequent to the acute onset of myofibre necrosis in young mice, a low level of necrotic and regenerating (recently necrotic) tissue, regenerated (with internal nuclei) and some unaffected (intact) myofibres can be observed in skeletal muscle from adult *mdx* mice. Necrotic, regenerating and regenerated muscles have distinct histological features [13]. *mdx* myofibres with internally located nuclei are a reliable indicator of previously necrotic/regenerated tissue. In older mice, Grounds *et al*. propose that the area of muscle that has not succumbed to necrosis exhibits a useful measure. The area of unaffected myofibres indicates resistance of the myofibres to damage. Unaffected myofibres look normal with peripheral nuclei.

In a joint effort supported by TREAT-NMD, histological parameters to improve histology based preclinical analysis were implemented (http://www.treat-nmd.eu/downloads/file/sops/). Currently, there exists no automated and standardised analysis system that is available to every laboratory. Analysis has to be carried out manually and therefore is biased to some degree.

Here, a globally accessible platform for histological analysis was developed. This platform allows, for example, comparability of histological evaluation and faster and more reliable preclinical drug candidate testing for DMD that can be applied to other degenerative neuromuscular disorders as well.

## Methods

### Mice

*Mdx* mice were obtained from Charles River Laboratories, Brussels, Belgium. The wild type control mice C57BL/10ScSnOlaHsd were purchased from Harlan Laboratories, Blackthorn, UK. Male mice were sacrificed at the age of 6 month by cervical dislocation. All animal experiments were carried out according to the German Animal Protection Law. The experiments were approved and controlled by the responsible authorities (55.2-1-54-2531-131-06, Regierung von Oberbayern, respectively Staedtisches Veterinaeramt, Munich, Germany).

### Histochemistry and immunohistochemistry

*M. tibialis anterior* (TA), *M. soleus* (Sol) and diaphragm (DIA) muscles were collected from 6 month old male C57BL/10 wild-type mice and *mdx* mice, respectively. Each group consisted of ≥ 7 mice (control group: n = 7, *mdx*: n = 8). Left and the right muscles of the hind limb were used. The whole muscle CSA was imaged and analysed in order to obtain less biased results. Altogether, about 2000 fibres were analysed for TA, about 3000 for DIA and 700 for Sol. Muscles were mounted on cork supports using gum tragacanth (Sigma-Aldrich, Steinheim, Germany). The samples were snap-frozen in isopentane cooled with liquid nitrogen and stored at −80°C.

The following procedure involves the use of immunological and histochemical reactions, which result in double staining for fast and slow myosin. Additionally, fluorescence staining of the myofibre membranes allows defining the minimal Feret’s diameter. The staining of myonuclei with the fluorescence dye bisbenzimide H (Hoechst 33258, Sigma-Aldrich) was utilised to identify nuclei located internally within myofibres.

Eight μm serial cross-sections of the muscle of interest were cut at a cryostat temperature of −25°C. We recommend using gelatin coated slides to ensure proper attachment of muscle sections during sequential staining. Slides were cleaned in 70% ethanol (Carl Roth GmbH, Karlsruhe, Germany) for 10 min and rinsed well in de-ionised water for 20 min. 500 ml of a 0.5% gelatin solution (Sigma-Aldrich) containing 0.05% chromium potassium sulphate (Sigma-Aldrich) was prepared at 60°C and filtered through standard filter paper. Slides were incubated in gelatine solution at 37-40°C for about 20 sec. Slides were allowed to dry overnight in a dust free incubator at a maximal temperature of 37°C. Until slides are used, it is recommended to store them at −20°C. Sections can be stored at – 20°C up to a few months.

After one hour at room temperature, sections were covered with 0.1% Triton X-100 (Sigma-Aldrich) in PBS (Phosphate buffered saline; PAA Laboratories GmbH, Pasching, Austria) solution, pH 7.3 for 15 min. Triton-X was removed and the slides were allowed to drain for a few minutes and were then arranged in humid chamber. A 1:100 dilution of slow myosin antibody (Novocastra, Newcastle upon Tyne, UK) in FCS (PAA Laboratories GmbH, Pasching, Austria) was applied to the sections and incubated at 4°C overnight. On the next day, the slow myosin antibody was rinsed off with 0.1% Triton X-100 in PBS and then washed in 0.1% Triton X-100 in PBS for an additional 30 min with one buffer change. Afterwards, the sections were covered with the secondary rabbit anti mouse IgG horseradish peroxidase (DAKO, Glostrup, Denmark) diluted 1:100 in 0.1 M lysine (Sigma-Aldrich), 40% Foetal Calf Serum (FCS) in PBS for 90 min at room temperature. Again, the antibody was rinsed off and sections were washed in 0.1% Triton X-100 in PBS for 30 min with one buffer change refreshing the solution after 15 min. Sections were covered with DAB (3,3’-diaminobenzidine tetrahydrochloride, Sigma-Aldrich) Peroxidase Substrate Solution [5 drops of 1% DAB (20x) added to 5 ml PBS, then mixed with 5 drops of 0.3% H_2_O_2_ (20x)] and incubated for 10 min. The DAB (20x) Peroxidase Substrate Solution was prepared as follows. 0.1 g of DAB was added to 10 ml distilled water. 3–5 drops of 10 N HCl (Carl Roth GmbH) were added until the solution turned light brown and mixed for 10 min. Aliquots were stored at −20°C.

Afterwards, sections were rinsed several times in tap water, rinsed briefly in PBS and incubated in FCS for 10 min. Without washing, the second primary antibody against fast myosin (NovoCastra), diluted 1:100 in FCS, was applied and incubated for 60 min. It is important to mention, that we obtained identical results when we interchanged the primary antibodies for slow and fast myosin. Next, the antibody was removed and slides were washed as described before. The sections were incubated with the same secondary antibody mentioned before together with 100 μg/ml Alexa Fluor 488 conjugated WGA (wheat germ agglutinin; Life Technologies GmbH, Darmstadt, Germany). After washing, the sections were covered with Vector SG visualisation solution (Vector Laboratories, Burlingame, CA, USA) for 10 min. 3 drops of chromogen were mixed with 3 drops of hydrogen peroxide substrate. The slides were washed in PBS for 5 min, followed by an incubation in 10 ng/ml Hoechst 33258 (bisbenzimide H) in PBS (50 μl each slide) for 5 min. Afterwards, slides were rinsed in PBS for 2–5 min, followed by a short rinse in distilled water. Nuclear counter stain was performed in Carazzi’s haematoxylin for 30 sec, blued up in running tap water for 2 min and mounted in mounting medium (DakoCytomation Fluorescent Mounting Medium, Dako). We utilised the nuclear counter stain to allow for double-check if nuclei were correctly identified. Carazzi’s hematoxylin solution was prepared as follows. 0.2 g hematoxylin (Merck KGaA, Darmstadt, Germany) was dissolved in distilled water at 50°C. The solution was cooled to room temperature and 0.04 g NaIO_3_ (Sigma-Aldrich), 10 g AlK(SO_4_)_2_ · 12 H_2_O (Sigma-Aldrich), and 40 ml glycerol (Merck) was added. Images were acquired using a Zeiss Axiovert 200 M fluorescence microscope and a Zeiss AxioCam HR photo camera. Images were taken at 20x magnification.

To register for the analysis service, the user is required to contact S.CO LifeScience GmbH (Munich, Germany) to get access to a personal web portal, which provides the MyoScan analysis module. The user is asked to purchase a volume package for this module type, which defines the number of analyses available. The web based analysis service is accessible worldwide any time. Currently, the costs for the analysis of one specimen are 5–10 €, depending on the prepaid volume package size. Technically, there is no limitation for the file size, apart from general limitations for upload processes via the world wide web. However, if a single file exceeds 20 MB the provider should be informed to ensure proper data handling. All common image file formats including .tif, .jpg, and .gif are supported. The maximum upload file number is limited by the size of the volume package the user has purchased.

### Quantitative assessment of muscle histology

Automatic quantitative analysis of muscle histology was performed with a specially designed module MyoScan for the web-based image analysis system S.CORE by S.CO LifeScience GmbH (http://www.sco-lifescience.com/technology.php5). Three different images of the observed section in the histological slice were taken: 1. Green fluorescent WGA staining for display of the myofibre boundaries, 2. Blue fluorescent Hoechst 33258 staining for display of the nuclei, 3. MHC (myosin heavy chain)-double staining for display of all myofibres which are positive for myosin slow type heavy chain or myosin fast type heavy chain, respectively. In the first image, all membrane structures were separated from background, leading to a mask for the individual muscle myofibres. This mask was superimposed first with the Hoechst 33258 image to identify the nuclei within myofibres resulting in a second mask displaying the relevant nuclei.

Both masks were superimposed with the MHC-double staining image to merge all relevant information in a single image (membrane: white, nuclei: black, MHC-double staining: colored). Based on the Cognition Network Technology of Definiens AG, Munich, relevant structures were extracted from the image as single objects and assigned to different object classes. This allows a detailed analysis of the relevant quantitative and morphometric data, e.g. number of nuclei per fibre and minimal Feret’s diameter.

Finally, each myofibre was automatically annotated with an ID and all data summarised in an Excel file, both as single data for each myofibre and as mean values. The variance coefficient (VC) was determined using the following formula:

$$\mathrm{VC}=1000\times \frac{\text{standard deviation of muscle myofibre minimal Feret diameters}}{\text{mean muscle myfibreminimal Feret's diameter}}\text{.}$$

Importantly, high image quality is essential to ensure optimal analysis. Therefore, folds, freezing artefacts, uneven or pale staining should be avoided as far as possible. Intensive, high quality staining of the myofibre boundaries is especially important. In our study labeling with WGA worked very well. However, any other membrane or extracellular matrix marker may be used instead. Nevertheless, the user is able to check each individual image processing step generated by MyoScan since result files are provided by the analysis system for individual processing if needed. Thus, the user can identify regions of the sections that are not appropriate for analysis. It is always possible to exclude any inappropriate data from analysis. Moreover, MyoScan applies an algorithm which screens generated images whether identified fibers fit the form and size compared to threshold parameters previously defined by the user.

To verify the data generated by MyoScan, manual analysis was carried out on whole muscle sections of TA muscle (n = 4) of 6 month old *mdx* mice using ImageJ software IJ 1.46r [14]. All sections were analysed for both minimal Feret’s diameter and internally nucleated fibres.

### Statistical analysis

Statistical analysis was performed using SPSS Statistics 17.0 software (SPSS GmbH Software, Munich, Germany) and MedCalc for Windows, version 12.3.0.0 (MedCalc Software, Mariakerke, Belgium) for comparison of the data generated by MyoScan with the manual analysis of muscle sections. All data sets were tested for normal distribution using the Kolmogorov–Smirnov test. Those data subsets that showed a normal distribution were analysed by Student’s *t*-test.

## Results

### Quantitative assessment of histological parameters in muscle sections of wild-type and *mdx* mice

**Figure 1:**
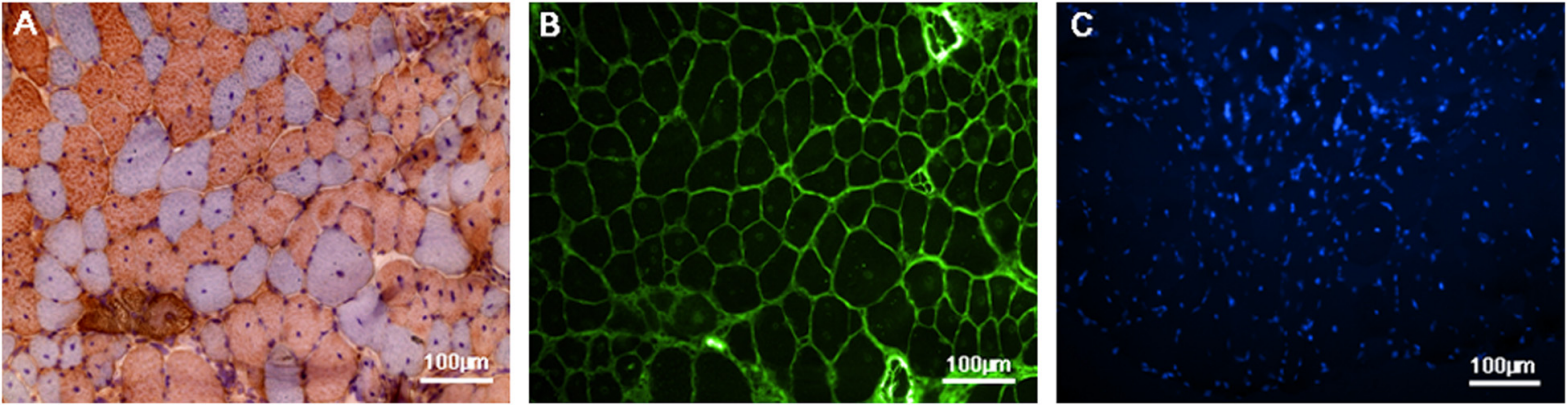
**Representative MHC-double staining.** (**A**) Myosin heavy chain (MHC)-double staining of a *M. soleus* (Sol) section obtained from a *mdx*-mouse. Slow myosin heavy chain fibres are displayed in brown and fast myosin heavy chain fibres are blue. (**B**) Wheat germ agglutinin staining (WGA staining) of the same section as in (**A**). WGA binds to *N*-acetylglucosamine and sialic acid residues at the myofibre membrane. (**C**) Nuclear staining with Hoechst 33258 of the same section as in (**A**) and (**B**).

**Figure 2:**
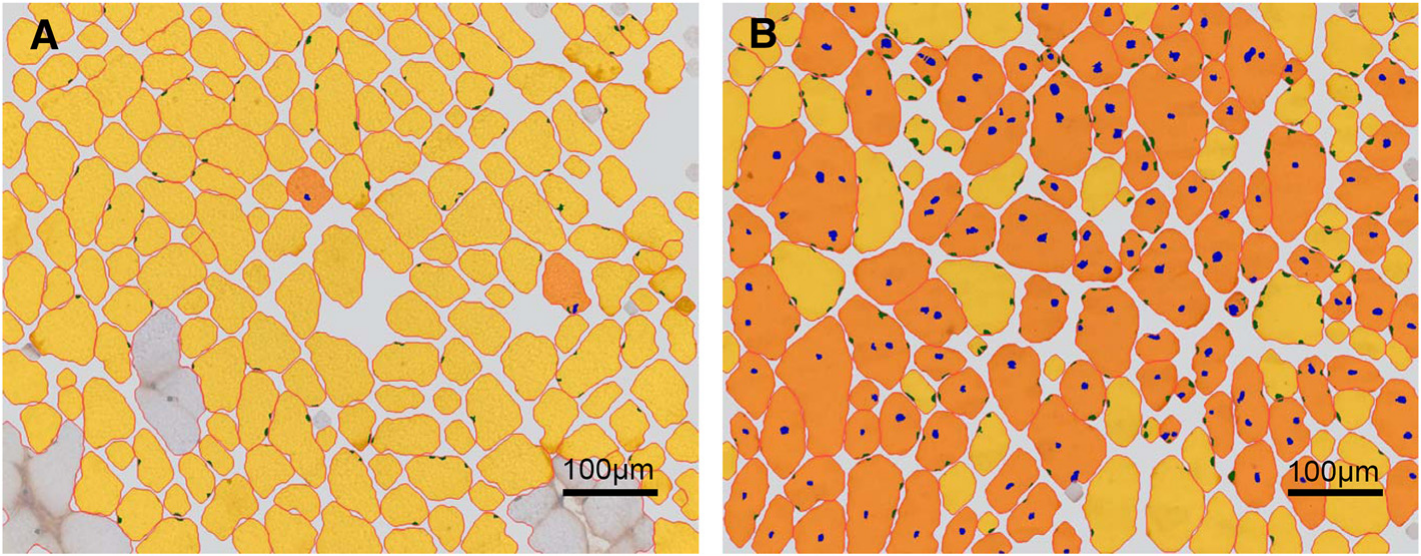
**Representative images processed by the S.CORE module “MyoScan” without annotations.** (**A**) Image of a 6 months old male C57BL/10 mouse. (**B**) Image of a 6 months old male *mdx* mouse. Yellow areas identify myofibres with peripheral nuclei (green dots), orange areas mark myofibres with internalised and internal nuclei (blue dots). Each myofibre is automatically annotated with an ID and all data is summarised in an Excel file (data not shown).

Percentage of muscle fibres with internal nuclei, mean minimal Feret’s diameter, variance coefficients and fibre size distribution were determined in three well-characterised muscles of wild-type and *mdx* mice: the diaphragm (DIA), *M. soleus* (Sol) and *M. tibialis anterior* (TA). These parameters were measured at 6 months of age on muscle section staining. Figure 1 shows representative images of a *M. soleus* section of a male *mdx* mouse immunodetected by MHC-double staining. While Figure 1A displays the staining with antibodies against myosin slow (brown fibres) and fast (blue fibres) type heavy chain, Figure 1B represents the staining of the fibre membrane with wheat germ agglutinin and Figure 1C shows the staining of the myonuclei with Hoechst 33258 on the same section. Figure 2 shows representative, processed images of a 6 months old male C57BL/10 mouse (Figure 2A) compared to an age-matched male *mdx* mouse (Figure 2B). Yellow areas indicate myofibres with peripheral nuclei (green dots), orange areas indicate myofibres with internalised nuclei (blue dots). Finally, numbers, which identify the myofibres’ ID, are automatically annotated. After analysis, resulting data are summarised in an Excel file and can be used for further statistical analysis. The provided data set comprises the image with and without annotations. The first histological parameter we determined was the percentage of muscle fibres with internalised nuclei.

### Significantly higher percentage of myofibres with internally located nuclei in *mdx* mice

**Figure 3:**
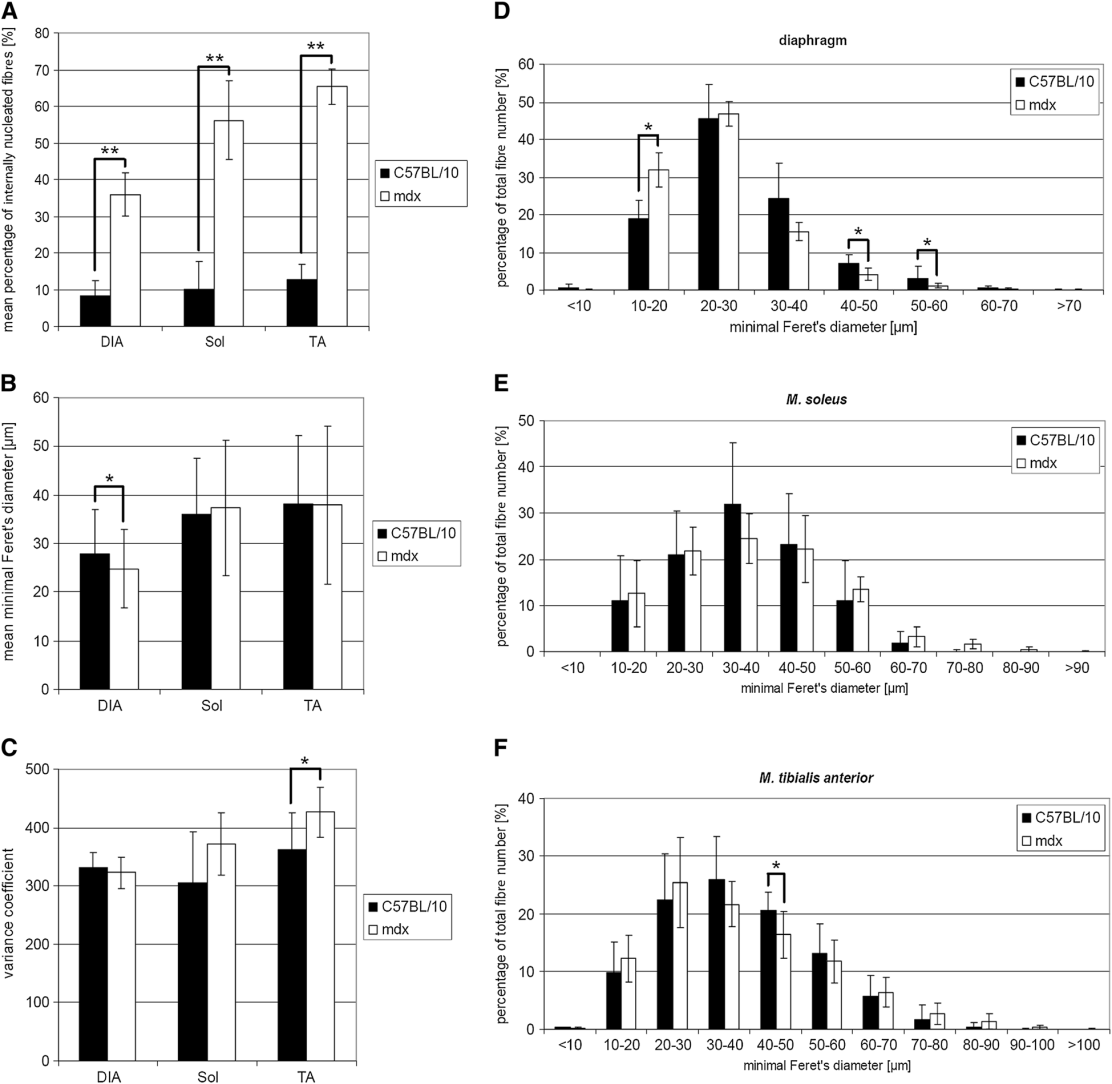
**Histological parameters in wild type and *mdx *mice.** (**A**) Percentage of muscle fibres with internalised nuclei determined in the diaphragm, *M. soleus* and *M. tibialis anterior* of wild type (black bars) and *mdx* mice (white bars) at 6 month of age. (**B**) Mean minimal Feret’s diameter determined in the diaphragm, *M. soleus* and *M. tibialis anterior* of wild type (black bars) and *mdx* mice (white bars) at 6 month of age. (**C**) Mean variance coefficient determined in the diaphragm, *M. soleus* and *M. tibialis anterior* of wild-type (black bars) and *mdx* mice (white bars) at 6 month of age. (**D**-**F**) Fibre size distribution determined as percentage of the total fibre number in the diaphragm (**D**), *M. soleus* (**E**) and *M. tibialis anterior* (**F**) of wild-type (black bars) and *mdx* mice (white bars) at 6 month of age. Data are shown as mean ± SD; n ≥ 7 mice; 1300–4300 myofibres per group; *p < 0.05, **p < 0.001 unpaired *t*-test.

Myofibres of DIA, Sol and TA with internalised nuclei were rarely seen in C57BL/10 wild-type mice. They never represented more than 13% of the total number of muscle fibres analysed (Figure 3B). Compared to the wild-type mice, DIA, Sol and TA muscles of *mdx* mice contained 35.94 ± 5.93%, 56.23 ± 10.82% and 65.35 ± 4.83% myofibres with internalised nuclei, respectively (Figure 3A).

### The mean minimal Feret’s diameter is significantly reduced in diaphragm of *mdx* mice

The minimal ‘Feret’s diameter’ is the morphometric parameter that changes the least with the orientation of the sectioning angle and thus was used to avoid biased results. The minimal Feret’s diameter is the most robust parameter in order to measure muscle fibre size and to determine the fibre size variance coefficient (VC) compared to other parameters like area, minimal inner diameter or perimeter [12, TREAT-NMD SOP (ID) number: DMD_M.1.2.001].

The mean minimal Feret’s diameter of wild-type mice yielded 27.87 ± 9.23 μm (DIA), 36.0 ± 11.48 μm (Sol) and 38.11 ± 14.03 μm (TA) while *mdx* mice revealed a mean fibre size of 24.82 ± 8.07 μm (DIA), 37.36 ± 13.91 μm (Sol) and 37.96 ± 16.28 μm (TA) (Figure 3B). The difference in mean minimal Feret’s diameter between the diaphragm of wild-type and *mdx* mice is significant (p = 0.003). This observation is indicative of a higher number of smaller fibres, which is characteristic for newly formed muscle fibres during repeated cycles of regeneration and degeneration as well as for atrophic fibres [8].

### *M. tibialis anterior* and *M. soleus* reveal an increased variance coefficient in *mdx* mice

Dystrophic muscles display a high degree of variability in the myofibre size because of a higher number of smaller fibres and the occurrence of hypertrophic fibres. This parameter is highly sensitive and is used routinely for detecting differences between dystrophic and healthy muscles [12].

Muscles of *mdx* mice showed a significantly higher variance coefficient in TA muscle compared to age-matched wild-type mice (C57BL/10: 362.10 ± 62.70, *mdx*: 426.72 ± 42.84, p = 0.035) (Figure 3C). In Sol of *mdx* mice, we observed a higher variance coefficient not significantly different to wild-type mice (C57BL/10: 305.02 ± 87.04, *mdx*: 371.04 ± 53.53) (Figure 3C). By contrast, variance coefficients in diaphragm are very similar (C57BL/10: 330.74 ± 26.43, *mdx*: 322.74 ± 26.97.53) (Figure 3C).

### In *mdx* mice a shift towards a higher number of smaller myofibres was observed

The analysis of the fibre size distribution revealed a tendency of a shift towards a higher number of smaller myofibres in *mdx* mice compared to C57BL/10 mice. While the fibre size distribution in Sol was very similar in both groups (Figure 3E), DIA and TA showed significant changes (Figure 3D and F). In DIA *mdx* mice had significant more myofibres below average size in a range of 10 to 20 μm than wild-type mice (C57BL/10: 19.05 ± 4.64%, *mdx*: 32.00 ± 4.57%, p = 0.0001). At the same time, the number of larger myofibres in the range between 30 and 50 μm were significantly reduced. C57BL/10 showed a percentage of 24.40 ± 9.24% fibres in the range of 30 to 40 μm and *mdx* 15.55 ± 2.47% (p = 0.02). In the range of 40 to 50 μm, C57BL/10 mice showed a percentage of 7.00 ± 2.44% fibres and *mdx* mice 4.16 ± 1.67% (p = 0.02). In TA, a significant decrease of the number of myofibres was observed only in the range of 40 to 50 μm (C57BL/10: 20.63 ± 3.13%, *mdx*: 16.36 ± 4.06%, p = 0.04). In TA of *mdx* mice, the number of smaller myofibres between 10 and 20 μm were not significantly increased (C57BL/10: 9.86 ± 5.31%, *mdx*: 12.22 ± 4.05%) and the number of fibres in the range between 30 and 40 μm (C57BL/10: 25.93 ± 7.40%, *mdx*: 21.63 ± 3.94%) were not significantly reduced as compared to C57BL/10 mice (Figure 3F).

### Bland-Altman analysis of the parameters minimal Feret’s diameter and the percentage of fibres with internalised nuclei determined manually and generated by MyoScan

**Figure 4:**
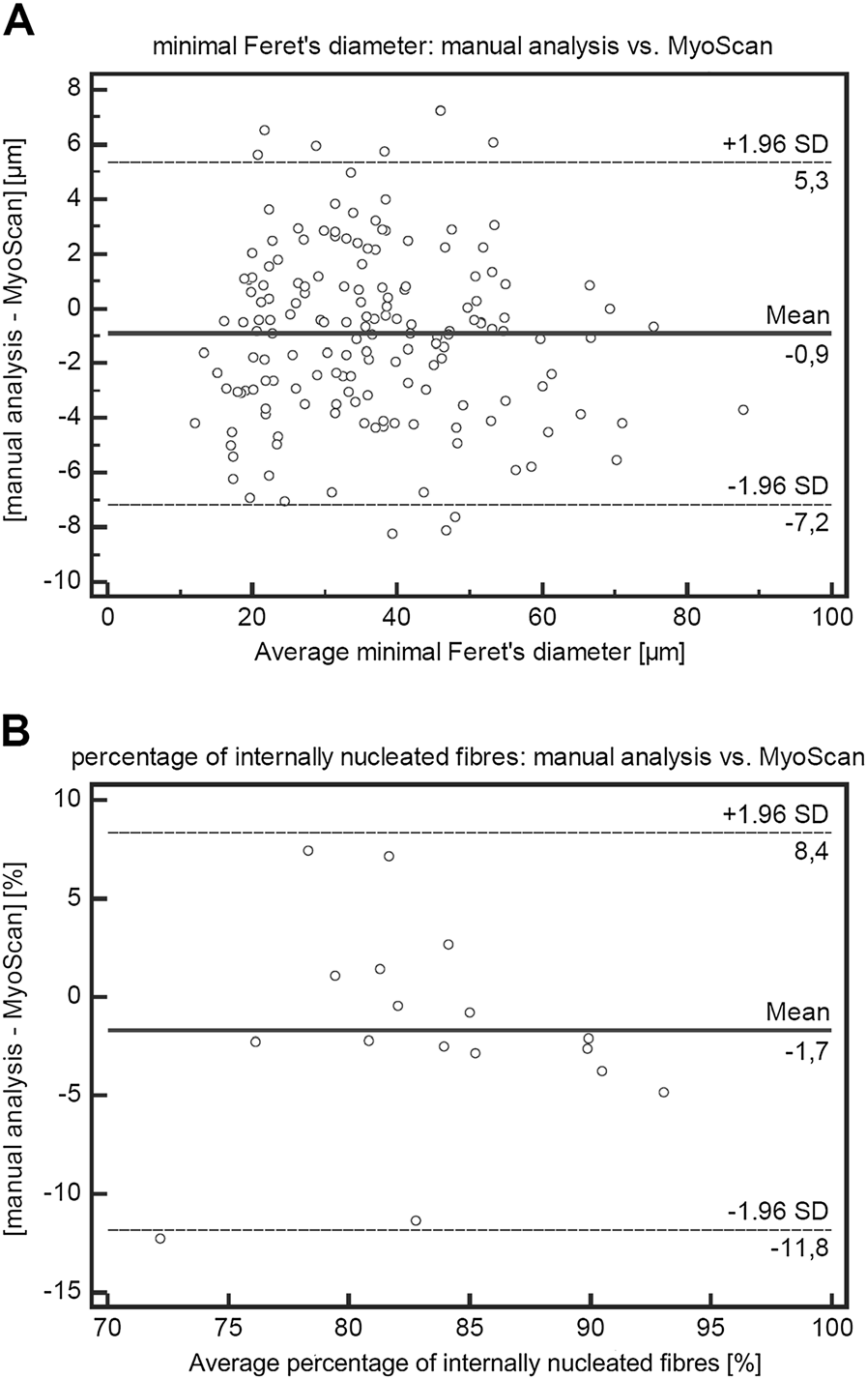
**The Bland-Altman procedure confirms equivalence between manual and MyoScan analysis.** Methodological comparison of measurement of minimal Feret’s diameter (**A**) and percentage of internally nucleated myofibres (**B**).

Results obtained from manual analysis of whole muscle sections of TA muscle (n = 4) of 6 month old *mdx* mice were compared to results received form automatic analysis of the same muscle sections using MyoScan. To this end, we generated a Bland-Altman plot [15-17] for both parameters, minimal Feret’s diameter (Figure 4A) and the percentage of fibres with internalised nuclei (Figure 4B) using the MedCalc software program. The plot displays the average of the paired values from each method on the x-axis and the difference of each pair of readings on the y-axis. The Bland-Altman plot is recommended to assess the relative agreement between two analytical methods [16]. The mean difference in values obtained with the two methods is called the bias [17]. Method comparison of the parameter minimal Feret’s diameter yields a bias of −0.9 μm (Figure 4A) and regarding the parameter percentage of internally nucleated fibres we observed a bias of −1.7% (Figure 4B).

The limits of agreement represent the range of values in which there is agreement between both methods for approximately 95% of the values [17]. For the minimal Feret’s diameter, the Bland-Altman plot displays the upper limit of agreement at 5.3 μm and the lower limit at −7.2 μm (Figure 4A). For the determination of internalised nuclei, Figure 4B shows an upper limit of agreement at 8.4% and a lower limit of agreement at −11.8%, whereas about 76% of the sample are below 5.0% and are located close to the line indicating the bias of −1.7%.

## Discussion

We performed simultaneous fluorescence staining of the myofibre membrane by fluorescence dye conjugated wheat germ agglutinin and of the nuclei by Hoechst 33258. Thus, we determined the percentage of internally nucleated fibres and the fibre size variance coefficient. In order to evaluate regeneration and necrosis, it is important to consider the age of the *mdx* mice as different histological features change with age [13]. Myofibres with internal nuclei are a reliable indicator of previously necrotic/regenerated tissue. Though, internal nuclei cannot indicate how often a muscle has undergone necrosis and subsequent regeneration. Rather, the area of muscle that was not affected by necrosis represents the favoured measure in studies of older mice, since this indicates resistance of the myofibres to damage.

In this study, we validated the MyoScan analysis module by comparing wild-type C57BL/10 mice with *mdx* mice, since our results were similar to earlier findings in the literature [11,12]. C57BL/10 mice showed hardly any internally nucleated fibres in contrast to *mdx* mice, where we identified up to 65% fibres with internal nuclei. Louboutin et al. (1993) reported a percentage of about 40% internally nucleated myofibres in diaphragm of 120 and 270 days old *mdx* mice, a value similar to our findings (35.94 ± 5.93%). In TA and Sol they observed about 70% and 65% compared to 65.35 ± 4.83% and 56.23 ± 10.82% in our study. A difference of 10% in the percentage of internally nucleated fibres in Sol compared to previous findings in the literature and the higher standard deviation of 10.82% may be due to the fact, that only 700 fibres were analysed in Sol muscle since the CSA of this muscle is smaller than that of DIA or TA.

Moreover, the fibre size distribution analysed in DIA and TA revealed the same shift towards a greater number of smaller myofibres in the range of 10 to 20 μm and a loss of larger myofibres in *mdx* mice compared to control mice as was found in our study [11]. Concerning the fibre size variance coefficient, wild-type mice presented lower values when compared to *mdx* mice. In TA, a significant higher variance coefficient was observed in *mdx* mice. These findings are compatible with the assumption that in *mdx* mice subsequent regeneration causes the appearance of newly regenerated myofibres in which nuclei remain in an internal location for a few months and of a higher variability of fibre sizes [12]. In addition to comparing our data with previous findings in literature, we conducted a methodological comparison between manual analysis and the MyoScan method in TA muscle sections (n = 4) of 6 month *mdx* mice using the Bland-Altman procedure.

The mean difference in values obtained with the two methods is called the bias [17]. Biases for both parameters, the minimal Feret’s diameter and the percentage of internally nucleated fibres, are relatively close to zero (Figure 4) confirming that automated and manual analysis generate very similar results. The Bland-Altman plot for the percentage of internally nucleated fibres shows a slightly higher bias than for the Feret’s diameter (Figure 4B). However, the difference is not large enough to be clinically important, since the discrepancy between both methods is less than 5.0% for about 76% of the samples. The biases and limits of agreement for both parameters are within a very small range that we assume to be acceptable for nonclinical applications. In conclusion, both methods are equally well suited to evaluate the analysed parameters. Since we examined only 6 month old mice, additional analysis in *mdx* mice of different age will be required. The automatic MyoScan analysis tool has already been successfully applied in a recent study where the efficacy of carboanhydrase inhibitors for treatment of a DMD model was assessed [18]. The method revealed a reduction of internally nucleated fibres in *M. tibialis anterior of mdx* mice treated with carboanhydrase inhibitors and allowed to identify changes of the fibre size variation [18]. The efforts of the TREAT-NMD consortium to set standards in preclinical development and animal models for the development of new therapeutic treatments in neuromuscular disorders resulted in the publication of guidelines and implementation of histological parameters for the *mdx* mouse [13,19]. An initial set of recommended histopathological parameters was implemented in MyoScan, which is able to be upgraded and represents an automatic image analysis system available on a platform for objective and quantitative histological analysis of muscle sections. Here, we provide protocols for a standardised and automated method to assess the percentage of fibres with internalised nuclei and the variance coefficient. Moreover, the degree of fibrosis represents an additional parameter, which can be used to specify potentially beneficial treatment. Furthermore, assessing the fibre type distribution might be of interest. The described MHC-double staining provides all necessary information for the analysis of these parameters and allows an upgrade of the developed method. Any colour change is dependent on the proportion of each MHC isoform. While co-expression of slow and fast myosin is detectable by double staining, very low levels of co-expression may not be detectable. In order to identify hybrid fibres with low levels of co-expression, we suggest separating the MHC-double staining described here. MHC staining may be done separately for slow or fast myosin while still assessing the percentage of fibres with internalised nuclei and the variance coefficient.

Despite the fact that muscle pathology for dystrophin deficiency differs between the *mdx* mouse model and human patients, the *mdx* mouse model is the most important animal model for preclinical studies in DMD research [19,20]. In addition to the outcome measures discussed here, the histological extent of fibrosis or functional analysis like muscle force measurements provide essential data to assess the dystrophic phenotype. Furthermore, histological changes in the *mdx* mouse model are often used as a surrogate marker for potential therapeutic benefit. The determination of fibre size variability is challenging. Consequently, the quantitative assessment of the fibre size variability is rarely performed. In this study, we validated a standardised and mainly automated quantitative assessment of histopathological parameters in the *mdx* mouse model that provides rapid and unbiased results.

## Conclusions

The analysis of dystrophic pathology on histological muscle sections is highly interpretative and thus can vary between individuals and laboratories. Moreover, a statistically sound and secure histological analysis of muscle sections entails an intense technical and time-consuming effort. Requirements for that kind of analysis are not provided in each laboratory on a regular basis. Therefore, a standardised and mainly automated quantitative assessment of histopathological parameters in the *mdx* mouse model is desirable to allow an objective comparison between laboratories. The developed method allowed us to perform a histological analysis of pathological changes of muscle fibres, particularly of the percentage of internally nucleated fibres and of the fibre size variance coefficient determined by the Feret’s diameter. Those parameters describe the process of degeneration and regeneration in *mdx* mice. The web-based image analysis system S.CORE with its specially designed module MyoScan offers a convenient solution, because it is generally available for all laboratories and enables an automated quantitative analysis of both parameters. The automated analysis system MyoScan used in this study is not limited exclusively to dystrophin-deficient mice, but also represents a useful tool for applications in the research of other dystrophic pathologies and in various other skeletal muscle diseases.

## Abbreviations

DAB: 3,3’-diaminobenzidine tetrahydrochloride; H 33258: Bisbenzimide H; DIA: Diaphragm; DMD: Duchenne Muscular Dystrophy; FCS: Foetal calf serum; MHC: Myosin heavy chain; PBS: Phosphate buffered saline; Sol: *M. soleus* ; TA: *M. tibialis anterior* ; VC: Variance coefficient; WGA: Wheat germ agglutinin.

## Competing interests

Dr. Markus Eblenkamp is CEO at the S.CO LifeScience GmbH, Munich, Germany. We developed the analysis platform S.CORE in close cooperation with S.CO LifeScience GmbH. Parts of the analysis were conducted by S.CO LifeScience for a fee. All other authors declare no competing interest.

## Authors’ contributions

ME, SP and EW developed the module MyoScan for the web-based image analysis system S.CORE. AP carried out the immunochemistry and histochemistry. CP performed the statistical analysis. CT conceived the study, and participated in its design and coordination and helped to draft the manuscript. CP, ME and CT participated in the design of the study. SK, HL and MCW participated in interpretation of data and have been involved in drafting the manuscript and gave final approval of the version to be published. All authors read and approved the final manuscript.

## Pre-publication history

The pre-publication history for this paper can be accessed here:

http://www.biomedcentral.com/1471-2474/14/26/prepub
